# Pro- and Anti-inflammatory Effects of High Cholesterol Diet on Aged Brain

**DOI:** 10.14336/AD.2017.0706

**Published:** 2018-06-01

**Authors:** Yali Chen, Mengmei Yin, Xuejin Cao, Gang Hu, Ming Xiao

**Affiliations:** Jiangsu Province Key Laboratory of Neurodegeneration, Nanjing Medical University, Nanjing, China; Jiangsu Province Key Laboratory of Neurodegeneration, Nanjing Medical University, Nanjing, China; Jiangsu Province Key Laboratory of Neurodegeneration, Nanjing Medical University, Nanjing, China; Jiangsu Province Key Laboratory of Neurodegeneration, Nanjing Medical University, Nanjing, China; Jiangsu Province Key Laboratory of Neurodegeneration, Nanjing Medical University, Nanjing, China

**Keywords:** aging, brain, high cholesterol diet, neuroinflammation, synaptic proteins

## Abstract

Both hypercholesterolemia and aging are related to cognitive decline or Alzheimer’s disease. However, their interactive influence on the neurodegenerative progress remains unclear. To address this issue, 6-month-old and 16-month-old female mice were fed a 3% cholesterol diet for 8 weeks, followed by hippocampus-related functional, pathological, biochemical and molecular analyses. The high cholesterol diet did not exacerbate age-dependent cognitive decline and hippocampal neuronal death, and even greatly mitigated decreases of synaptophysin and growth associated protein 43 expression in the hippocampus of aged mice. Compared with young controls, aged mice fed normal diet showed mild activation of hippocampal microglia with increased expression of CD68, a marker of the microglial M1 phenotype, and decreased expression of CD206, a marker of the microglial M2 phenotype. More interestingly, the high cholesterol diet not only improved NLRP3 inflammasome activation and IL-1β expression, but also increased levels of anti-inflammatory cytokines IL-4 and IL-6 in the hippocampus of old mice, suggesting playing pro- and anti-neuroinflammatory effects. In addition, the cholesterol rich diet resulted in a defect of the blood-brain barrier of aged hippocampus, as revealed by increased brain albumin content. These results have revealed both harmful and protective effects of high cholesterol diet on aged brain, which helps us to understand that hypercholesterolemia in the aged population is not associated with dementia and cognitive impairment.

Cholesterol plays a key role in maintaining physical, chemical and functional properties of the lipid bilayer membrane [1] However, persistent hyper-cholesterolemia is associated with a variety of diseases, such as atherosclerosis, coronary heart disease, diabetes and stroke [2-4] Moreover, hypercholesterolemia could cause neuroinflammation, thereby increasing the risk of age-related neurological disorders including Alzheimer’s disease (AD) [5-7] For instance, a 30-year retrospective cohort study revealed that hypercholesterolemia in midlife is associated with a 27% increase in risk of late-life dementia [8] Nevertheless, the evidence that serum cholesterol levels as a risk factor for dementia and cognitive impairment remains inconclusive, partly attributable to different classes of lipoprotein exert different or even opposite regulatory roles in the peripheral and central nervous system (CNS) inflammation and oxidative stress [9] Cholesterol is essential for maintaining normal brain functions, such as signal transduction, synaptic plasticity, and memory formation [10] Even though the CNS accounts for only 2.1% of body weight, it contains 23% of total body cholesterol, which is much higher than other peripheral tissues [11] Nearly all the brain cholesterol is synthesized de novo and separated from the peripheral cholesterol by the brain-blood-barrier (BBB) [11] But it does not mean that the dietary intake of cholesterol cannot affect the cholesterol metabolism in the CNS In contrast to cholesterol itself, several side-chain hydroxylated metabolites of cholesterol, such as 24-hydroxycholesterol and 27-hydroxycholesterol, are able to cross the BBB Both of these hydroxycholesterols are involved in the neuroinflammation [12, 13] But on the other hand, aged brain needs a greater demand for cholesterol due to age-related cholesterol loss [14] Therefore, a cholesterol-enrich diet in the elderly could have a protective effect on the brain function [15], which in turn offsets its negative consequence, especially on the neuroinflammation This hypothesis is supported by epidemiological evidence that hypercholesterolemia has no effect on dementia and cognitive impairment in the elderly [16], and lower serum cholesterol level in the aged populations even increases the risk of AD [17] In spite of this, there is a lack of pathological and molecular evidence for this conclusion.

In order to define the internal interaction between aging and high cholesterol diet in the development of brain pathology, in the present study, 6-month-old and 16-month-old female mice were fed a 3% cholesterol diet for 8 weeks, then hippocampus-related spatial cognitive function, synaptic protein levels, glial reactivation, neuroinflammation and pro-inflammatory signal transduction pathway were analyzed.

## MATERIALS AND METHODS

### Animals and experimental design

Six-month-old and sixteen-month-old female CD1 mice were divided into four groups (n =12-14 per group): young-normal-diet (young ctrl) and aged-normal-diet group (aged ctrl), fed a normal diet; young-high-cholesterol-diet (young HC) and aged-high-cholesterol-diet (aged HC), fed one containing 3% cholesterol for 8 weeks. The diet components are described previously [18]. Mice were housed under controlled temperature and photoperiod conditions (12 h light/dark), with food and water available *ad libitum*. Body weight was measured once a week. All experiments were conducted in accordance with international standards on animal welfare and the guidelines of the Institute for Laboratory Animal Research of Nanjing Medical University. All efforts were made to minimize reduce the number of animals used.

### Y-maze test

The Y maze test was performed to measure mouse short-term memory, as previously described [19]. Briefly, during the training stage, the novel arm was blocked by a black baffle, and mouse was allowed to move freely only in the other two arms for 8 min. During the testing stage, the novel arm was opened, and the mouse could freely move throughout all 3 arms for 5 min. The percentage of time spent in the novel arm and the numbers of entries were calculated.

### Morris water maze (MWM)

The MWM task was performed to measure mouse long-term spatial learning and memory function [20]. Training was conducted over 6 consecutive days, with 4 trials per day. During the first 2 days, mice were trained to find a dark-colored visible platform with a diameter of 10 cm and 0.5 cm above the water surface. On the 3rd day, the platform was moved to the opposite quadrant and submerged 1 cm below the water surface. The escape latency and swimming speed were analyzed. On the 7st day, the hidden platform was removed, allowing mice to swim in the pool for 60 s. The percentage of total time spent in the target quadrant and the platform crossing times were counted. The above activities of mice were collected by a digital video camera connected to a computer-controlled system (Beijing Sunny Instruments Co. Ltd, China). All tests were performed by two independent experimenters, who were each blind to the experimental schedule.

### Blood and brain tissue sample preparation

The mice were deeply anesthetized with 3.5% chloralhydrate (1ml/100g, i.p.). Body weight was measured, and blood was collected from the orbital vein. The blood samples were centrifuged at 3000 rpm for 30 min at 4? in a microcentrifuge, and then the serum was collected and stored at -20? until ready for cholesterol assay. For Western blotting, mice were decapitated, and the hippocampus was rapidly dissected out and stored at -80?. For pathological analysis, mice were transcardially perfused with 0.9% saline followed by 4% paraformaldehyde. The brains were then removed, post-fixed overnight at 4? dehydrated in a series of graded ethanol solutions and then embedded in paraffin. The coronal sections were serially cut at 5 μm using a paraffin slicing machine (LeicaRM2135, Nussloch, Germany).

### Serum cholesterol analysis

The enzymatic colorimetric kits (Kexin Biotechnology Institute, Shanghai, China) were used to detect the serum total cholesterol (TC), high-density lipoprotein cholesterol (HDL-C) and low-density lipoprotein cholesterol (LDL-C) on Hitachi 7250 Automatic analyzer (Tokyo, Japan) [21]. All samples were analyzed in duplicate.

### Nissl staining

The deparaffinized sections were stained in 0.5% cresyl violet solution for 30 min,then differentiated in 70% ethyl alcohol with 2-3 drops of 10% acetic acid. After differentiation, slides were dehydrated in ethyl alcohol, cleared in xylene, and mounted with neutral resin for microscopic examination.

### Immunohistochemistry

The deparaffinized tissue sections were incubated with mouse monoclonal anti-glial fibrillary acidic protein (GFAP) antibody (1:800; Millipore, USA), rabbit polyclonal anti-ionized calcium-binding adaptor molecule 1 (Iba-1) antibody (1:1000; Wako, Japan) or rabbit polyclonal anti-cysteine-aspartic acid protease 3 (caspase-3) (1:1000; Cell Science Technology, USA) at 4? overnight [18]. After washing with PBS, sections were incubated with biotinylated-conjugated goat anti-mouse or rabbit IgG (1:200; Vector Laboratories) for 1 h at 37? and visualized by DAB (Sigma Aldrich, USA).

### Cell counting and image analysis

The hippocampal section images were captured by a digital microscope (Leica Microsystems, Wetzlar, Germany). Degenerated neurons are characterized by perikaryal cytoplasmic contraction and affinity for cresyl violet, and often vacuolation of adjacent neuropil [21]. The percentage of degenerated neurons in the hippocampal CA1 was calculated. Caspase-3 positive CA1 neurons were also counted. The percentage of neuronal apoptosis was expressed. GFAP or Iba-1 immunostaining in the hippocampus was semi-quantified as previously described [18, 22]. Briefly, GFAP or Iba-1-immunopositive cells with complete cellular profile were hand recorded, and expressed as the number of cells per mm^2^. The area of each GFAP or Iba-1-immunopositive cells was also measured using an Image-Pro Plus 6.0 Analysis System (Media Cybernetics Inc., USA). Four sections per mouse, and 4 mice per group, were used to provide a mean value for each index mentioned above.

### Quantitative real-time PCR

Total RNA was extracted from hippocampal tissues with Trizol reagent (Invitrogen Life technologies, USA). Reverse transcription PCR was carried out using a TaKaRa Prime Script RT reagent kit and real-time PCR was measured using a QuantiTect SYBR Green PCR kit (Qiagen, USA) with an ABI 7300 Fast Real-Time PCR System (Applied Biosystems, USA). cDNA was amplified and quantified by regular PCR and real-time PCR. The following primer sequences were used: interleukin-1β (IL-1β) (forward: 5′-CGTCTCCCAGA GCCAATCC-3′; reverse, 5′-CACCAGGCTGACTTT GAGTGAGT-3′); IL-4 (forward: 5′-GGTCTCAACC CCCAGCTAGT-3′; reverse: 5′-GCCGATGATCTCT CTCAAGTGAT-3′); IL-6 (forward: 5′-ATCCAGTT GCCTTCTTGGGACTGA-3′; reverse: 5′-TAAGCCTCC GACTTGTGAAGTGGT-3′); IL-10 (forward: 5′-GCT CTTACTGACTGGCATGAG-3′; reverse: 5′-CGCAG CTCTAGGAGCATGTG-3′); tumor necrosis factor (TNF-α) (forward: 5′-CATCTTCTCAAAATTCGAGT GACAA-3′; reverse: 5′-TGGGAGTAGACAAGGTAC AA-3′); GAPDH (forward: 5′-CAAAAGGGTCATCT CC-3′; reverse: 5′-CCCCAGCATCAAAGGTG-3′). PCR thermal cycle parameters were as follows: 95? for 10 min, 40 cycles of 60? for 60 s, and 95? for 15 s, and a melting curve from 60? to 95? to ensure amplification of a single product. The GAPDH gene was used as an endogenous control to normalize for differences in the amount of total RNA in each sample.

### Western blotting

Hippocampal tissues were homogenized and centrifuged at 4?, 12,000 rpm for 15 min. The protein samples were transferred onto PVDF membranes using a Bio-Rad miniprotein-III wet transfer unit, then blocked with 5% skimmed milk dissolved in TBST at room temperature for 1 h. After washing with TBST buffer three times, membranes were incubated at 4? overnight with one of the primary antibodies listed in Table 1. Peroxidase-conjugated goat anti-rabbit IgG, horse anti-rat IgG or horse anti-goat IgG (1:2000; all from Vector Laboratories) was used, and bands were visualized using ECL plus detection system. GADPH was utilized as an internal control for protein loading and transfer efficiency.

### Statistical analysis

All data were expressed as means ± SEM. The MWM platform training data were analyzed by repeated-measures ANOVA with day of training as the within-subject variable, and age and treatment (high cholesterol) as the between-subject factors. The other data were analyzed by two-way ANOVA with age and treatment as factors, followed by a Bonferroni correction for multiple comparisons. A value of *p*<0.05 was considered statistically significant.

**Table 1:** Antibodies used in the Western blotting.

Antibodies	kDa	Clone	Dilution	Source	Catalog number
Anti-mouse serum albumin ASC	69 24	Goat, polyclonal Rabbit, polyclonal	1:1000 1:500	Abcam Santa Cruz	ab19194 Sc-22514
Caspase 1 CD31 CD68 CD206 GAPDH IKKβ IL-1β NLRP3 p-IKKβ P65 p-P65 Procaspase 1 ProIL-1β PSD95 SYP ZO-1	20 130 68 206 37 88 17 106 88 65 65 46 34 85 70 220	Rabbit, polyclonal Goat, polyclonal Rat, FA-11 Goat, polyclonal Rabbit, polyclonal Rabbit, polyclonal Rabbit, polyclonal Rabbit, polyclonal Rabbit, polyclonal Rabbit, polyclonal Rabbit, polyclonal Rabbit, polyclonal Rabbit, polyclonal Rabbit, polyclonal Rabbit, polyclonal Rabbit, polyclonal	1:1000 1:2000 1:400 1:1000 1:2000 1:1000 1:500 1:1000 1:1000 1:1000 1:1000 1:1000 1:1000 1:1000 1:1000 1:1000	Millipore R&D Bio-Rad R&D Bioworld Cell Signaling Millipore Adipogen Cell Signaling Cell Signaling Cell Signaling Millipore Millipore Abcam Abcam Santa-cruz	06-503-I AF3628 MCA1957T AF2535 AP0063 8943 AB1832P AG-20B-0014-C100 2697 8242 3033 06-503-I AB1832P Ab18258 Ab64581 Sc-10804

**Figure 1.**
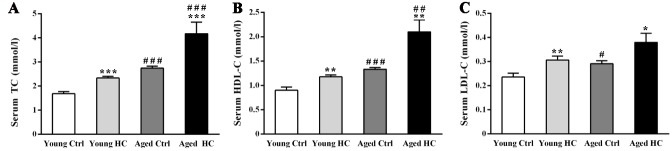
Serum cholesterol analysis of young and aged female mice fed a normal or high-cholesterol diet for 8 weeks. (A) Total cholesterol (TC). (B) High-density lipoprotein cholesterol (HDL-C). (C) Low-density lipoprotein cholesterol (LDL-C). Results were expressed as mean ± SEM. Tests were performed in duplicate on 8 serum samples per group. *P < 0.05, **P < 0.01, ***P < 0.001, high cholesterol diet (HC) mice versus normal control (ctrl) diet mice; #*P* < 0.05, ##*P* < 0.01, ###*P* < 0.001, aged mice versus young mice.

## RESULTS

### Both high-cholesterol diet and aging increased serum TC, HDL-C, and LDL-C levels

The serum levels of TC, HDL-C, and LDL-C were detected to exam the effectiveness of hyper-cholesterolemia model (Fig. 1A-C). Two-way ANOVA revealed significant effects of age [F_TC (1,28)_ = 33.912, P < 0.001; F_HDL-C (1,28)_ = 26.784, P < 0.001; F_LDL-C (1,28)_ = 7.719, P = 0.01], treatment [F_TC (1,28)_ = 17.539, P < 0.001; F_HDL-C (1,28)_ = 15.905, P < 0.001; F_LDL-C (1,28)_ = 11.735, P < 0.001], but not their interaction [F_TC (1,28)_ = 2.447, P = 0.129; F_HDL-C (1,28)_ = 3.494, P = 0.072; F_LDL-C (1,28)_ = 0.164, P = 0.689]. TC and HDL-C levels in aged HC mice were higher than those of young HC mice (P < 0.001 and P < 0.01, respectively).

**Figure 2.**
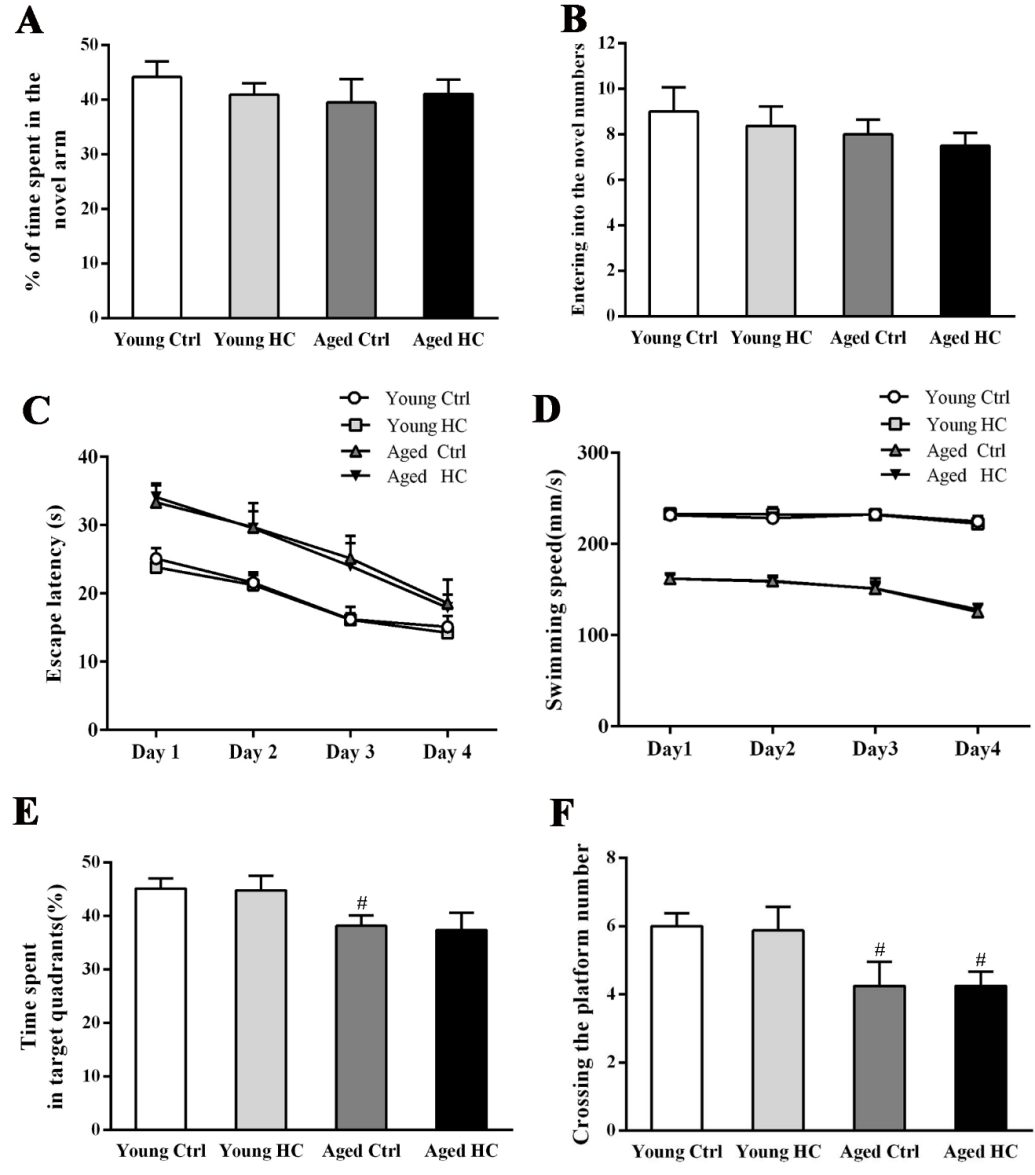
Cognitive analysis of young and aged female mice fed a normal or high-cholesterol diet for 8 weeks. (A) The percentage of time spent in the novel arm of the Y-maze. (B) The numbers of entries into the novel arm of the Y-maze. (C) The mean escape latency during the hidden platform training period of the Morris water maze test. (D) Swimming speed. (E) The percentage of time spent in the target quadrant. (F) The number of crossing the platform area. Results were expressed as mean ± SEM from 8 mice per group. #*P* < 0.05, aged mice versus young mice.

**Figure 3.**
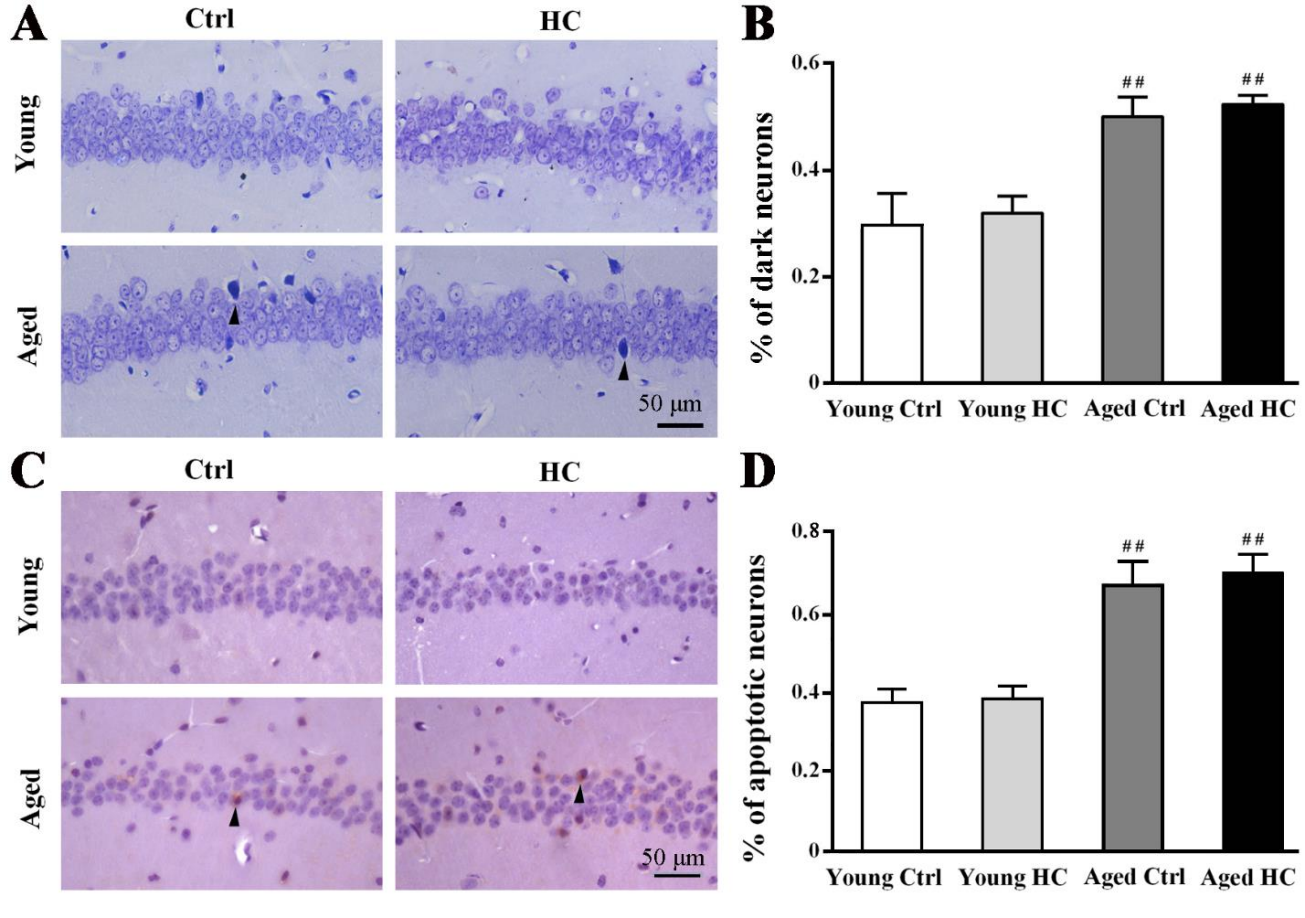
Pathological analysis of hippocampal neurons of young and aged female mice fed a normal or high-cholesterol diet for 8 weeks. (A) Nissl staining. Degenerative (dark) neurons were densely stained (arrowheads). (B) The percentage of dark neurons. (C) Immunostaining for cleaved-caspase 3. A few apoptotic neurons were marked by arrowheads. (D) The percentage of cleaved-caspase 3 positive neurons. Results were expressed as mean ± SEM of tests in duplicate on 4 hippocampal samples per group. ##*P* < 0.01, aged mice versus young mice.

### High cholesterol diet did not exacerbate the spatial cognitive deficits of aged mice

The mouse working memory was assessed by the Y-maze testing. Two-way ANOVA revealed that the percentage of time spent in the novel arm and entering into the novel arm numbers were not affected by age [F_time spent (1,28)_ = 0.554, P = 0.463; F_entering numbers (1,28)_ = 1.331, P = 0.258], treatment [F_time spent (1,28)_ = 0.079, P = 0.780; F_entering numbers (1,28)_ = 0.479, P = 0.494] and their interaction [F_time spent (1,28)_ = 0.612, P = 0.441; F_entring numbers (1,28)_ = 0.006, P = 0.939] (Fig. 2A, B). The spatial learning and memory of mice was measured using the MWM test. Repeated-measures ANOVA revealed significant effects of age [F_escape latency (3,112)_ = 54.672, P < 0.001; F_swimming speed (3,112)_ = 581.306, P < 0.001], but not of treatment [F_escape latency (3,112)_ = 1.445, P = 0.239; F_swimming speed (3,112)_ = 0.088, P = 0.769] or their interaction [F_escape latency (3,112)_ = 0.002, P = 0.963; F_swimming speed (3,112)_ = 0.013, P = 0.910] (Fig. 2C, D). The percentage of time spent in the target quadrant and the numbers crossing the platform were affected significantly by age [F_time spent (1,28)_ = 11.672, P = 0.002; F_crossing numbers (1,28)_ = 5.939, P = 0.021], but neither by treatment [F_time spent (1,28)_ = 0.229, P = 0.636; F_crossing numbers (1,28)_ = 0.278, P = 0.602] nor their interaction [F_time spent (1,28)_ = 0.268, P = 0.609; F_crossing numbers (1,28)_ = 0.275, P = 0.603] (Fig. 2E, F).

### High cholesterol diet did not increase neuronal degeneration and apoptosis in the hippocampus of aged mice

The degenerated neurons in the hippocampal CA1 was examined by Nissl staining (Fig. 3A). Two-way ANOVA revealed significant effects of age [F_(1,12)_ = 13.361, P = 0.003], but not of treatment [F_(1,12)_ = 0.333, P = 0.575] or their interaction [F_(1,12)_ = 0.000, P = 1.0] (Fig. 3B). Correspondingly, age [F_(1,12)_ = 10.949, P = 0.006], but not treatment [F_(1,12)_ = 0.091, P = 0.768] or their interaction [F_(1,12)_ = 0.001, P = 0.979], had significant effects on the percentage of caspase-3 positive neurons in the hippocampal CA1 (Fig. 3C, D).

**Figure 4.**
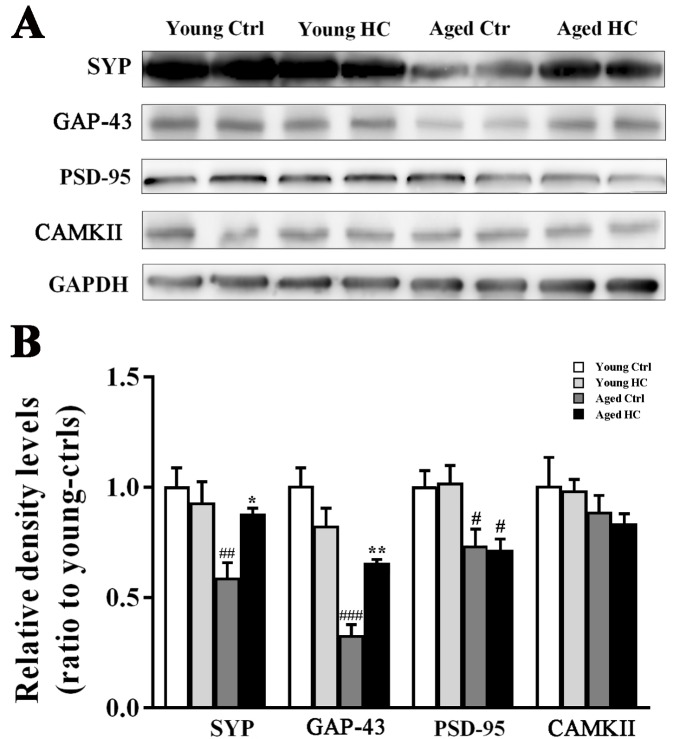
Analyses of synapse-related protein levels in the hippocampus of young and aged female mice fed a normal or high-cholesterol diet for 8 weeks. (A) Representative immunoblot bands of SYP, GAP43, PSD-95 and CAMKII. (B) The corresponding densitometry analysis. Results were expressed as mean ± SEM of tests in duplicate on 4 hippocampal samples per group. *P < 0.05, **P < 0.01, high cholesterol diet (HC) mice versus normal control (ctrl) diet mice; #*P* < 0.05, ## *P* < 0.01. ### *P* < 0.001, aged mice versus young mice.

**Figure 5.**
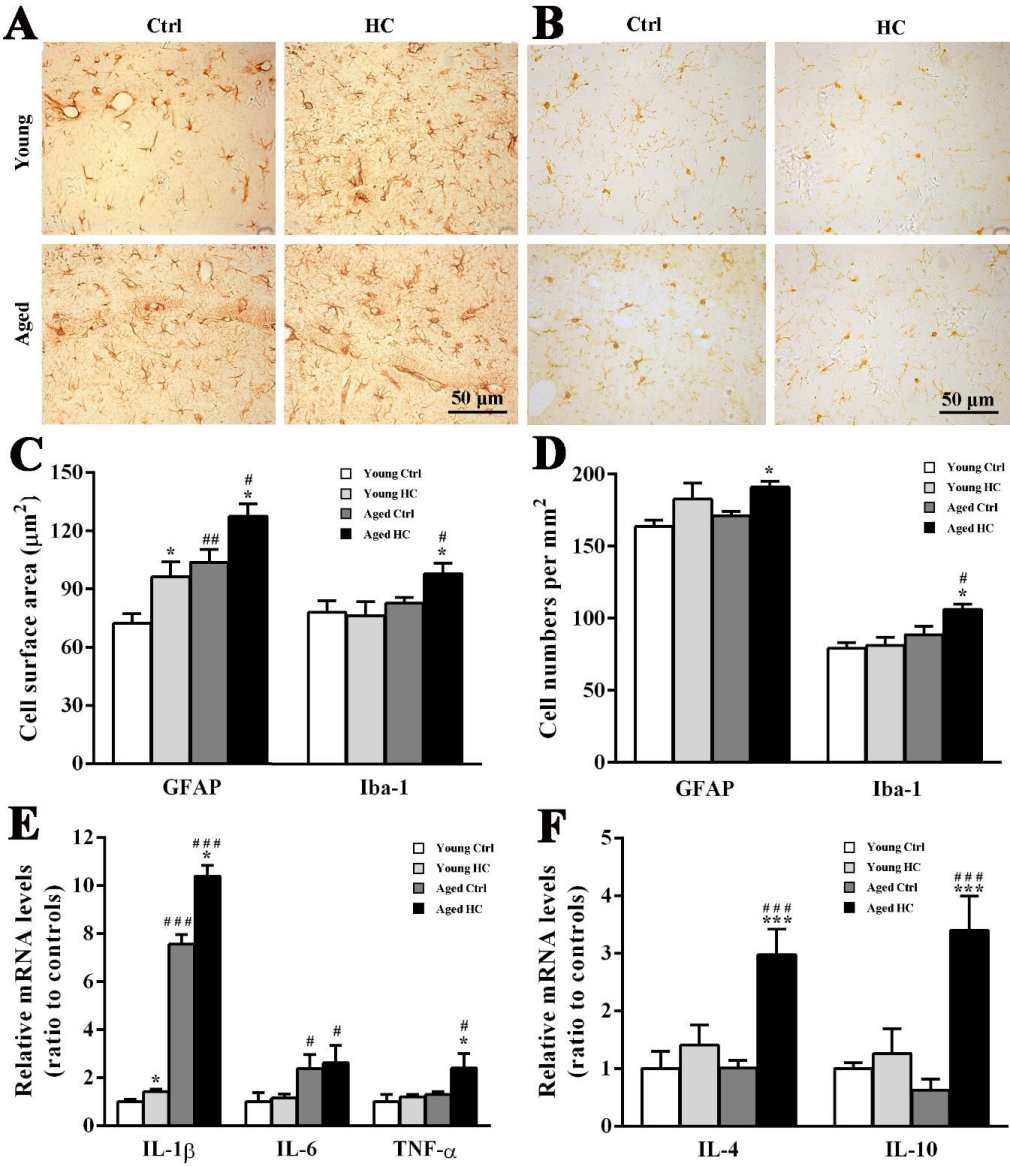
Analyses of glial activation and levels of pro-inflammatory and anti-inflammatory factors in the hippocampus of young and aged female mice fed a normal or high-cholesterol diet for 8 weeks. (A) Immunohistochemical staining for GFAP. (B) Immunohistochemical staining for Iba-1. (C) Cellular surface of GFAP positive astrocytes and Iba-1 positive microglia. (D) Cell counts of GFAP positive astrocytes and Iba-1 positive microglia. (E) The quantitative real-time PCR analysis of mRNA levels of IL-1β, IL-6 and TNF-α. (F) The quantitative real-time PCR analysis of mRNA levels of IL-4 and IL-10. Results were expressed as mean ± SEM of tests in duplicate on 4 hippocampal samples per group. *P < 0.05, ***P < 0.001, high cholesterol diet (HC) mice versus normal control (ctrl) diet mice; #*P* < 0.05, ###*P* < 0.001, aged mice versus young mice.

**Figure 6.**
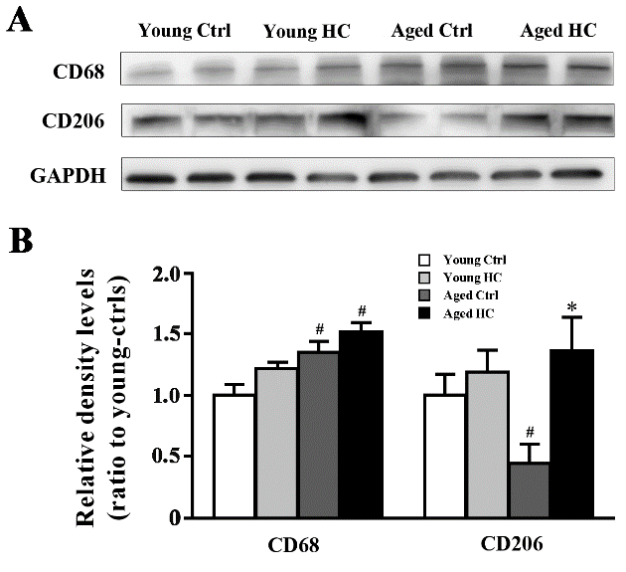
Levels of CD68 and CD206 in the hippocampus of young and aged female mice fed a normal or high-cholesterol diet for 8 weeks. (A) Representative immunoblot for CD68 and CD206. (B) The corresponding densitometry analysis. Results were expressed as mean ± SEM of tests in duplicate on 4 hippocampal samples per group. *P < 0.05, high cholesterol diet (HC) mice versus normal control (ctrl) diet mice; ^#^*P* < 0.05, aged mice versus young mice.

### High cholesterol diet increased presynaptic protein expression in the hippocampus of aged mice

In order to investigate the effect of high cholesterol diet on hippocampal synapses, we examined expression levels of presynaptic proteins, such as synaptophysin (SYP) and growth-associated protein-43 (GAP-43), as well as post-synaptic proteins including post-synaptic density protein-95 (PSD-95) and Ca^2+^/calmodulin-dependent protein kinase II (CaMKII) in the hippocampus of mice. Age affected the expression levels of SYP [F_(1,12)_ = 12.843, P = 0.004] and GAP-43 [F_(1,12)_ = 38.084, P < 0.001], but not of PSD-95 [F_(1,12)_ = 0.953, P = 0.348] and CAMKII [F_(1,12)_ = 1.395, P = 0.260]. Treatment had no effect on SYP [F_(1,12)_ = 3.424, P = 0.070], GAP-43 [F_(1,12)_ = 1.120, P = 0.311], PSD-95 [F_(1,12)_ = 0.001, P = 0.989] or CAMKII expression [F_(1,12)_ = 0.100, P = 0.757]. The interaction effect was significant on SYP [F_(1,12)_ = 5.410, P =0.038] and GAP-43 [F_(1,12)_ = 13.425, P = 0.003], but was no significantly on PSD-95 [F_(1,12)_ = 0.027, P = 0.872] or CAMKII [F_(1,12)_ = 0.024, P = 0.88]. The high cholesterol diet significantly increased SYP and GAP-43 in the aged hippocampus (both P < 0.05) (Fig. 4A, B).

### High cholesterol diet exerted both pro- and anti-inflammatory effects on the hippocampus of aged mice

It has been reported that hypercholesterolemia evokes glial cell activation and neuroinflammation [18, 21, 25, 26]. We determined whether there is age difference in glial inflammatory response induced by high cholesterol diet. Immunohistochemistry showed that GFAP positive astrocytes were activated in the hippocampus of HC mice no matter young or aged (Fig. 5A). Two-way ANOVA revealed significant effects of age [F_(1,12)_ = 23.216, P < 0.001] and treatment [F_(1,12)_ = 13.443, P < 0.003], but not their interaction [F_(1,12)_ = 0.000, P = 0.985] on GFAP positive cell surface area. The cell body area of astrocytes in young HC mice and aged ctrl mice was lager than that in young ctrl mice (both P < 0.05), but was lower than that in aged HC mice (both P < 0.05) (Fig. 5C). In addition, treatment affected the number of GFAP positive astrocytes significantly [F_(1,12)_ = 9.383, P = 0.010], but not age [F_(1,12)_ = 1.511, P = 0.243] or their interaction [F_(1,12)_ = 0.010, P = 0.924]. The high cholesterol diet increased the number of GFAP positive cells in aged mice (both P < 0.05), but not in young mice (P > 0.05) (Fig. 5D).

Microglia were activated by the high cholesterol diet as well (Fig. 5B). Two-way ANOVA revealed significant effects of age [F_cell surface area (1,12)_ = 5.527, P = 0.037; F_cell number (1,12)_ = 11.814, P = 0.005], but not treatment [F_cell surface area 1,12)_ = 1.516, P = 0.242; F_cell number (1,12)_ = 3.774, P = 0.076] or their interaction [F_cell surface area (1,12)_ = 2.284, P = 0.157; F_cell number (1,12)_ = 2.385, P = 0.148]. The cell surface area and number of iba-1 positive microglia in aged HC mice were more than aged ctrl mice (both P < 0.05) (Fig. 5C, D).

**Figure 7.**
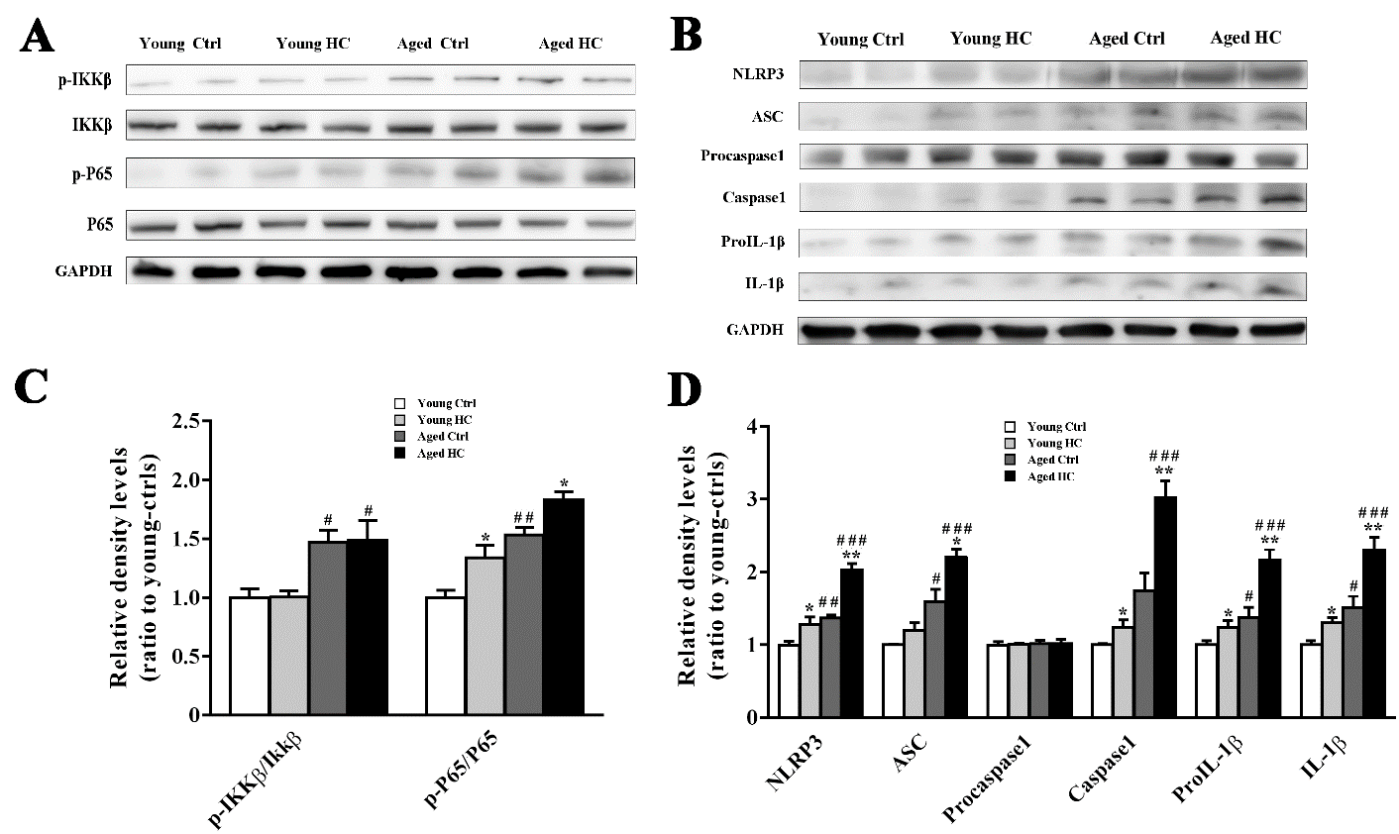
Activation of NLRP3 inflammasomes in the hippocampus of young and aged female mice fed a normal or high-cholesterol diet for 8 weeks. (A and B) Representative immunoblot of proteins involved in the first and second signal pathways of NLRP3 inflammasome activation. (C and D) The corresponding densitometry analysis. Results were expressed as mean ± SEM of tests in duplicate on 4 hippocampal samples per group. *P < 0.05, **P < 0.01, high cholesterol diet (HC) mice versus normal control (ctrl) diet mice; ^#^*P* < 0.05, ^##^*P* < 0.01, ^###^*P* < 0.001, aged mice versus young mice.

To determine neuroinflammatory consequences of glial activation, mRNA expression levels of pro-inflammatory cytokines IL-1β, TNF-α and IL-6, and anti-inflammatory cytokines IL-4 and IL-10 in the hippocampus were examined by quantitative real-time PCR. Age [F_(1,12)_ = 217.023, P < 0.001], treatment [F_(1,12)_ = 9.468, P = 0.01] and their interaction [F_(1,12)_ = 5.231, P = 0.041] had a signficant effect on IL-1β mRNA levels. Age [F_(1,12)_ = 4.940, P = 0.046] and treatment [F_(1,12)_ = 6.313, P = 0.027] also had a significant effect on TNF-α mRNA levels. Two-way ANOVA analysis showed significant effects of age [F_(1,12)_ = 9.834, P = 0.009], but not treatment [F_(1,12)_ = 0.077, P = 0.786] or their interaction [F_(1,12)_=0.021, P= 0.886] on IL-6 mRNA levels. Both IL-1β and TNF-α mRNA levels in the hippocampus of aged HC mice were higher than those of aged ctrl mice (both P < 0.05) (Fig. 5E).

In addition, treatment significantly affected mRNA levels of IL-4 [F _(1,12)_ = 10.270, P = 0.008] and IL-10 [F_(1,12)_ = 8.927, P = 0.011], while age [F_IL-4 (1,12)_ = 0.725, P = 0.411; F_IL-10 (1,12)_ = 0.653, P = 0.435] and their interaction [F_IL-4 (1,12)_ = 0.694, P = 0.421; F_IL-10 (1,12)_ = 2.398, P = 0.147] showed no significant effect. Aged HC mice had high mRNA levels of IL-4 and IL-10 compared with aged ctrl mice (both P < 0.05) or young HC mice (both P < 0.05) (Fig. 5F). Together, these results suggested that the high cholesterol diet results in both pro- and anti-inflammatory effects on aged mouse brain.

**Figure 8.**
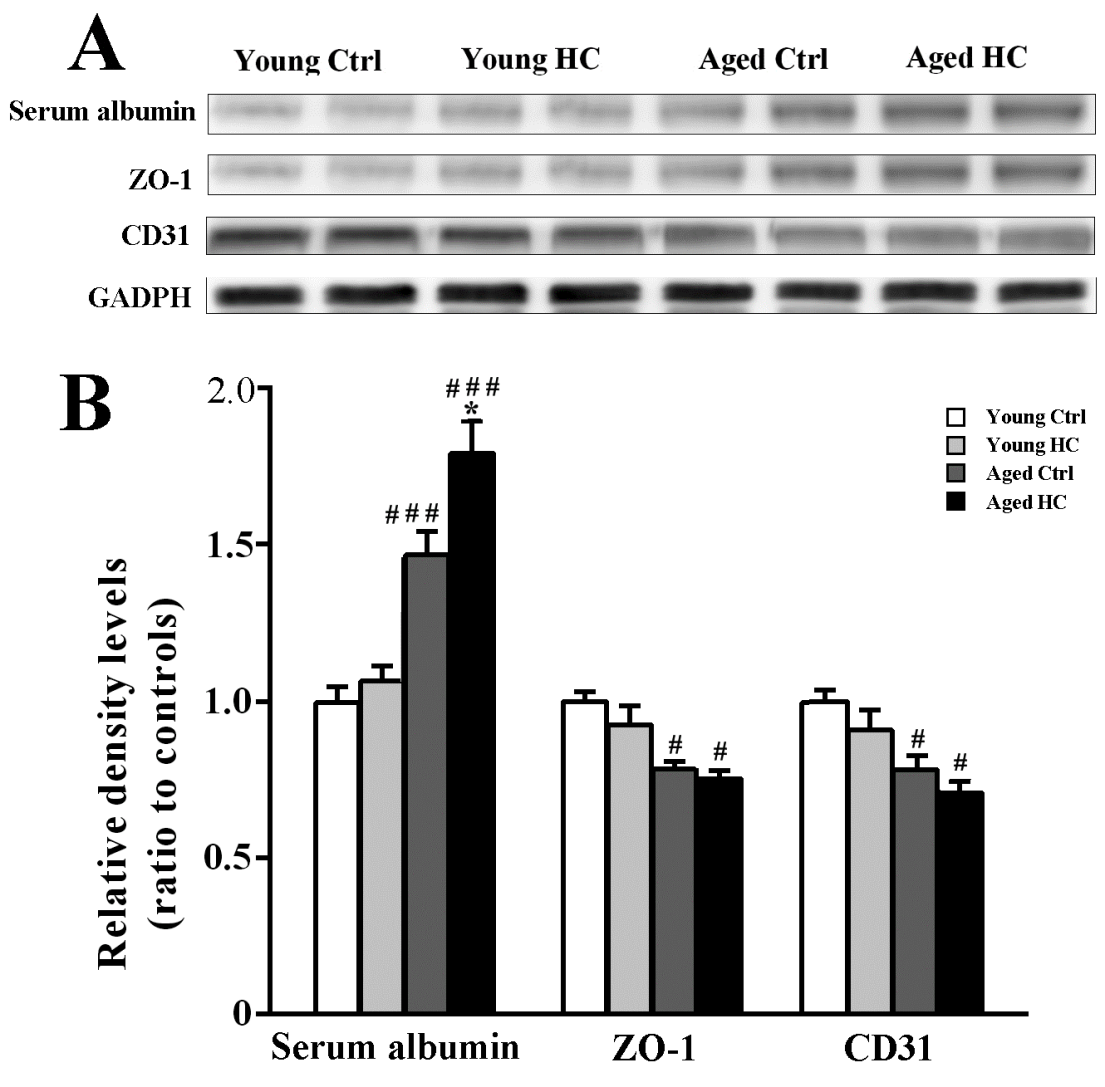
Analyses of BBB related protein levels in the hippocampus of young and aged female mice fed a normal or high-cholesterol diet for 8 weeks. (A) Representative immunoblot bands of ZO-1, serum albumin and CD31. (B) The corresponding densitometry analysis. Results were expressed as mean ± SEM of tests in duplicate on 4 hippocampal samples per group. *P < 0.05, high cholesterol diet (HC) mice versus normal control (ctrl) diet mice; ^#^*P* < 0.05, ^###^*P* < 0.01, aged mice versus young mice.

### High cholesterol diet promoted microglia polarization to M2 phenotype in the hippocampus of aged mice

Microglia play dual roles in the neuroinflammation, depending on their phenotypes [27, 28]. M1-like phenotype microglia express pro-inflammatory cytokines, such as IL-1β, TNF-α and IL-6. M2-like phenotype microglia are characterized by secretion of anti-inflammatory cytokines, such as IL-4 and IL-10, which could protect against inflammation by mitigating damages and enhancing tissue remodeling [29, 30]. To further investigate whether increases in brain anti-inflammatory cytokines in aged HC mice are associated with a high ratio of M2 microglia, we detected expression levels of CD68 and CD206 that could mark M1 and M2 microglia, respectively, in the hippocampus. The results revealed that age [F_(1,12)_ = 13.178, P = 0.003], but not treatment [F_(1,12)_ = 4.362, P = 0.059] or their interaction [F_(1,12)_ = 0.346, P = 0.567] affected the expression of CD68 significantly. Treatment [F_(1,12)_ = 10.897, P = 0.006], but not age [F_(1,12)_ = 1.232, P = 0.289] or their interaction [F_(1,12)_ = 4.370, P = 0.059] had a significant effect on the expression of CD206. Aged ctrl mice had high CD68 expression but low CD206 expression when compared to young ctrl mice (both P < 0.05), supporting the veiw that microglia switch from M2 to M1 phenotype during the ageing process [39]. However, high cholesterol diet totally reversed decreases in CD206 expression in aged mice (P < 0.01) (Fig. 6A, B).

### High cholesterol diet increased NLRP3 inflammasome activation in aged mouse hippocampus

We further explored the underlying mechanism for raised expression of IL-1β in the aged mouse hippocampus. In the central nervous system, the generation of bioactive IL-1β from activated glia is mainly mediated by the nucleotide binding oligomerization domain-like receptor containing a pyrin domain 3 (NLRP3) inflammasome [31]. NLRP3 is one type of inflammasomes whose assembly and activation involves a two-step process. Firstly, a priming signal stimulates toll-like receptor 4 and then enhances NF-κB-driven transcription of NLRP3. Then a second signal promotes NLRP3 to form a protein complex with apoptosis-associated speck-like protein containing a caspase recruitment domain (ASC) [32].

Western blot analysis revealed that age [F_p-IKKβ/t-IKKβ (1,12)_ = 18.710, P = 0.001; F_p-P65/t-P65 (1,12)_ = 41.449, P < 0.001] and treatment [F_p-IKKβ/t-IKKβ (1,12)_ = 0.013, P = 0.913; F_p-P65/t-P65 (1,12)_ = 16.079, P = 0.002], but not their interaction [F_p-IKKβ/t-IKKβ (1,12)_ = 0.003, P = 0.955; F_p-P65/t-P65 (1,12)_ = 0.076, P = 0.787] affected the p-IKKβ/t-IKKβ and p-P65/t-P65 expression ratios. There was a higher ratio of p-P65/t-P65 in the hippocampus of aged HC mice than aged ctrl mice (all P < 0.05) (Fig. 7A, C), suggesting that the high cholesterol diet increased the activation of NF-κB pathway.

Consistently, two-way ANOVA analysis revealed significant effects of age [F_NLRP3 (1,12)_ = 55.047, P < 0.001; F_Caspase1 (1,12)_ = 51.965, P < 0.001], treatment [F_NLRP3 (1,12)_ = 39.367, P < 0.001; F_Caspase1 (1,12)_ = 19.015, P = 0.001] and their interaction [F_NLRP3 (1,12)_ = 6.319, P = 0.027; F_Caspase 1 (1,12)_ = 8.751, P = 0.012] on the protein expression levels of NLRP3 and Caspase1. In addition, significant effects of age [F_ASC (1,12)_ = 47.396, P < 0.001; F_IL-1β (1,12)_ = 27.137, P < 0.001] and treatment [F_ASC (1,12)_ = 12.138, P = 0.005; F_IL-1β (1,12)_ = 14.261, P = 0.003], but not their interaction [F_ASC (1,12)_ = 3.255, P = 0.096; F_IL-1β (1,12)_ = 2.845, P = 0.117], on the ASC and IL-1β levels were observed (Fig. 7B, D). Together, these results suggest that high cholesterol diet further aggravates age-related NLRP3 inflammasome activation, subsequently enhancing IL-1β production.

### High cholesterol diet increased BBB permeability in aged mouse hippocampus

Both hypercholesterolemia and aging are related to a defective BBB [33, 34]. We examined brain albumin content, a marker for BBB permeability, and expression levels of BBB associated proteins zonula occludens-1 (ZO-1) and platelet endothelial cell adhesion molecule-1 (PECAM-1/CD31) in the hippocampus of each group [35-37]. Age had a significant effect on serum albumin content within the hippocampal tissue [F_(1,12)_ = 28.421, P < 0.001), and high cholesterol diet increased the brain albumin level in aged mice (P < 0.05), but not young controls (P > 0.05). This indicates a synergic effect of hypercholesterolemia and aging on increased permeability of the BBB. Age, but not high cholesterol diet, significantly decreased ZO-1 [F_(1,12)_ = 8.345, P = 0.014; F_(1,12)_ = 0.014, P = 0.640, respectively] and CD31 expression [F_(1,12)_ = 9.598, P = 0.009; F_(1,12)_ = 2.208, P = 0.163, respectively] in the hippocampus (Fig. 8A, B).

## DISCUSSION

The effect of cholesterol diet on neurodegeneration is controversial. A number of epidemiological studies have shown that hypercholesterolemia in midlife is related to an increased risk of developing AD or mild cognitive impairment in later life [8, 38]. However, other studies have suggested that high serum cholesterol levels are not associated with dementia and cognitive impairment in the elderly [16], and even decrease the risk of AD [39, 40]. But the underlying mechanisms for this discrepancy remain unclear. In order to clarify whether brain pathological changes induced by high cholesterol diet are age-differential, we have fed 6-month-old and 16-month-old female mice with the diet containing 3% cholesterol for 8 weeks, and examined corresponding changes in cognitive behavior, pathology and biochemistry.

We found that 8 weeks of a standard diet supplemented with 3% cholesterol increase serum TC, HDL-C and LDL-C in both young and aged mice, and aged HC mice have high levels of serum TC and HDL-C, but not LDL-C, compared to young HC mice. This result indicates that the high cholesterol diet used in the present study leads to a significant increase in TC, but primarily due to an increase in HDL, rather than in LDL. It has been reported that serum cholesterol is positively related to cognitive function in the old population, which may be due to a good standard of living and education levels [41]. The epidemiological studies have suggested that high levels of HDL-C in older women are related with better episodic memory ability and less conscious forgetting [42]. Increased HDL-C in a certain range might enhance the combination of free cholesterol and reduce lipid oxidative stress of microvascular endothelial cells, thus protecting the BBB integrity and CNS homeostasis [43]. Further study is necessary to investigate effects of aging factor on cognitive function of old mice fed atherogenic or Western-type diet, with higher levels of saturated fat and containing cholesterol to primarily increase serum LDL. The potential finding would contribute to clarifying overall effects of the diet with different types of lipoproteins on the aged brain.

It is well known that cognition functions, especially spatial reference memory, are impaired during physiological aging [44]. In the present study, we found that 18-month-old mice show poor performance of long-term spatial learning and memory in the MWM, but not of short-term working memory in the Y-maze tests. Our study also found that, compared with the normal diet group, 6-month-old mice received an eight-week of high cholesterol diet does not appear working and spatial memory dysfunction. This result is different from the previous studies reporting that a significant spatial cognitive impairment occurs in 4-month-old mice fed 2% cholesterol diet for 16 weeks [45]. These discrepant data suggest that cognitive deficits caused by hypercholesterolemia are chronic and progressive. And also, the aged mice fed high cholesterol diet spend similar time on finding the platform and similar residence time in the target quadrant, compared with aged ones fed the normal diet. Together, these results suggest that an eight-week of intake of 3% cholesterol diet is not sufficient to impair cognitive function of mice no matter they are young or old.

Consistent with cognitive decline, neuronal death and synaptic loss occur during the normal aging process [46, 47]. We found increases in degenerated or apoptotic neurons and decreases in SYP, GAP43 and PSD95 expression in the hippocampus of aged mice, compared with young ones. SYP is a synaptic vesicle membrane structural protein. GAP-43, a protein enriched in growth cones, is related to axonogenesis and synaptogenesis. They both belong to presynaptic proteins [48]. PSD-95 is a membrane associated guanylate kinase scaffolding protein located in the postsynaptic densities. CaMKII is also a marker for post-synaptic protein and critical for long-term potentiation and information storage [49]. In the present study, we found that 18-month-old mice have low levels of SYP and GAP-43 in the hippocampus compared with 8-month-old controls. However, PSD-95 and CaMKII levels are no different each other. Cholesterol is involved in the formation and plasticity of synapses [50, 51]. A certain amount of cholesterol supplementation may help to partially attenuate synaptic protein loss in the aged brain. In agreement with this view, we found that the age-related decreases of SYP and GAP-43 are greatly mitigated by the high cholesterol diet. By contrast, the high cholesterol diet did not affect PSD-95 and CaMKII expression in the hippocampus of both adult and aged mice. The underlying mechanism for differences in age/cholesterol-dependent synthesis of presynaptic proteins and post-synaptic proteins remains to be further explored.

Glia play an important role in maintaining the homeostasis of brain, such as phagocytosis and elimination of the harmful metabolites [52]. There is a close relation between astrocyte biology and cholesterol metabolism, since astrocytes are main cell sources for synthesis of cholesterol in the central nervous system [53]. Consistent with the previous study [18], in this study, we demonstrated that 6-month old mice fed diet containing 3% cholesterol for 8 weeks do not exhibit cognitive impairment, neuronal degeneration, synaptic protein loss and microglial activation, but do display mild astrogliosis in the hippocampus. This result supports with the view that astrocyte activation is an early and primary event in respond to a variety of chronic and progressive pathological changes and stresses [54].

Some studies suggest that activated microglia play a more critical role in the response to various stress and disease processes, compared with the early response of astrocytes [55,56]. According to different types and stages of pathological conditions, activated microglial cells also show different proportions between M1 microglia that are main resource of inflammatory cytokines and M2 microglia that have a neuroprotective effect via secretion of anti-inflammatory factors, respectively [27-30]. In this study, we found that feeding aged mice with normal diet results in activation of microglia with upregulated protein expression of M1 marker CD68, but downregulated expression of M2 maker CD206, compared with young controls. Consistently, increased IL-1β expression has been demonstrated in the gene and protein levels. This finding is consistent with the view that activated microglia display age-dependent phenotype [57]. Moreover, interestingly, we found that feeding aged mice with high cholesterol further augments microglial activation with upregulation of both CD68 and CD206 at protein levels as well as upregulation of inflammatory cytokines IL-1β, IL-6 and TNF-α and anti-inflammatory cytokines IL-4 and IL-10 at mRNA levels. The antagonism between anti-inflammation and pro-inflammation might ameliorate the deleterious roles of hypercholesterolemia in the late onset of neurodegeneration.

The activation of NLRP3 inflammasome comprises two main signals: the first signal is that when pathogen-associated pattern recognition receptors bind with ligands, NF-κB signaling pathway is activated that involves promotion of IL-1β precursor and transcription of NLRP3. The second signal is that mature caspase-1, ‘catalyzed’ by ASC, cleaves proIL-1β to mature IL-1 [31, 32]. In this study, we revealed that high cholesterol diet further enhances aged-related upregulation of these marker proteins of NLRP3 inflamma-some signaling pathway in the mouse hippocampus, subsequently contributing to a dramatic increase in IL-1β levels.

It is known that plasma oxycholesterols induce oxidative damage of microvascular endothelium cells, subsequently impairing the BBB [33]. In the present study, we showed that the high cholesterol diet increased albumin content within the hippocampal tissue of aged mice, but not young mice, suggesting a synergic effect of hypercholesterolemia and aging on increased permeability of the BBB. In addition, aged hippocampus had low levels of endothelial tight junction protein ZO-1 and adhesion molecule CD31, which are responsible for maintaining the integrity of BBB [36]. The disrupted BBB has been shown to increase influx of cholesterol into the brain and efflux of brain 24-hydroxycholesterol into the peripheral circulation [58]. Quantitative analyses of brain cholesterol including it hydroxylated metabolites are necessary to elucidate the interactive influences of hypercholesterolemia and aging on cholesterol metabolic disturbance in the CNS.

It should be noted that, except dual regulation of pro- and anti-inflammatory condition via controlling microglial phenotype in the aged brain, high cholesterol diet may also affect brain pathology via regulating the levels of steroid hormones. The sex hormones, especially estrogen, are well documented in their neuroprotective roles, including against neuroinflammation [59]. Because cholesterol is a precursor of steroid hormones, high cholesterol intake may increase the sex hormone synthesis, and attenuate age-related hormone insufficiency, subsequently exerting beneficial effects on the brain [60]. Accumulated epidemiological evidences reveal that estrogen replacement therapy is helpful to reduce the risk of AD [61]. Consistent with this, our previous studies reported that a high cholesterol diet for 9 weeks increases serum estradiol levels in ovariectomized mice and alleviates hippocampal spatial cognitive dysfunction and pathological damage caused by endogenous estrogen deprivation [21]. In addition, high cholesterol diet can affect the metabolism of Aβ in the brain through various ways including affecting the expression levels of several enzymes related with Aβ production and clearance [18, 62, 63]. Furthermore, β-secretase and γ-secretase increase and Aβ degradation enzymes decrease with age [64-67]. Therefore, age-dependent changes of Aβ metabolism-related enzymes might also be involved in the relation between high cholesterol diet and AD pathogenesis. All these issues need to be investigated in the future.

In summary, the present results have showed that a high cholesterol diet induced brain pathology is more complicated in aged mice than young controls. The high cholesterol diet increases NLRP3 inflammasome activation and IL-1β production, meanwhile it also reverses decrease of M2 microglia with upregulation of anti-inflammatory cytokines IL-4 and IL-10, thus playing both pro- and anti-inflammatory roles in aged brain. Notably, we found that the high cholesterol diet greatly mitigates decreases in SYP and GAP-43 levels in the aged hippocampus, which may also contribute to counteract neuroinflammation-induced synaptic impairment. It is worth noting that the above results are based on a period of 8 weeks of diet containing 3% cholesterol. A more comprehensive and systematic study is necessary by treatment with different content and duration of high cholesterol diet. All these studies would help us to further understand the relationship between abnormal cholesterol metabolism and neurodegenerative diseases.
